# Phenotypic and genotypic characterization of colistin-resistant *Escherichia Coli* with *mcr-4*, *mcr-5*, *mcr-6*, and *mcr-9* genes from broiler chicken and farm environment

**DOI:** 10.1186/s12866-023-03118-y

**Published:** 2023-12-08

**Authors:** Mulu Lemlem, Erkihun Aklilu, Maizan Mohamed, Nor Fadhilah Kamaruzzaman, Zunita Zakaria, Azian Harun, Susmita Seenu Devan, Intan Noor Aina Kamaruzaman, Mohd Farhan Hanif Reduan, Muthupandian Saravanan

**Affiliations:** 1 Faculty of Veterinary Medicine, Universiti Malaysia Kelantan, Kota Bharu, Kelantan 16100 Malaysia; 2 Department of Medical Microbiology and Immunology, College of Health Science, Mekelle University, 231, Mekelle, Tigray Ethiopia; 3 Faculty of Veterinary Medicine, Universiti Putra Malaysia, Serdang, Selangor 43400 Malaysia; 4 School of Medical Sciences, Universiti Sains Malaysia, Kota Bharu, Kelantan 15200 Malaysia; 5 AMR and Nanotherapeutics Lab, Department of Pharmacology, Saveetha Dental College, Saveetha Institute of Medical and Technical Sciences (SIMATS), Chennai, 600077 India

**Keywords:** AMR, Colistin resistance, *Escherichia coli*, *mcr*, Broiler chicken, Environment

## Abstract

**Background:**

Colistin is an antibiotic used as a last-resort to treat multidrug-resistant Gram-negative bacterial infections. Colistin had been used for a long time in veterinary medicine for disease control and as a growth promoter in food-producing animals. This excessive use of colistin in food animals causes an increase in colistin resistance. This study aimed to determine molecular characteristics of colistin-resistant *Escherichia coli* in broiler chicken and chicken farm environments.

**Results:**

Four hundred fifty-three cloacal and farm environment samples were collected from six different commercial chicken farms in Kelantan, Malaysia. *E. coli* was isolated using standard bacteriological methods, and the isolates were tested for antimicrobial susceptibility using disc diffusion and colistin minimum inhibitory concentration (MIC) by broth microdilution. Multiplex PCR was used to detect *mcr* genes, and DNA sequencing was used to confirm the resistance genes. Virulence gene detection, phylogroup, and multilocus sequence typing (MLST) were done to further characterize the *E. coli* isolates. Out of the 425 (94%; 425/453) *E. coli* isolated from the chicken and farm environment samples, 10.8% (48/425) isolates were carrying one or more colistin-resistance encoding genes. Of the 48 colistin-resistant isolates, 54.2% (26/48) of the *mcr* positive isolates were genotypically and phenotypically resistant to colistin with MIC of colistin ≥ 4 μg/ml. The most prominent *mcr* gene detected was *mcr-1* (47.9%; 23/48), followed by *mcr-8* (18.8%; 9/48), mcr-7 (14.5%; 7/48), *mcr-6* (12.5%; 6/48), *mcr-4* (2.1%; 1/48), *mcr-5* (2.1%; 1/48), and *mcr-9* (2.1%; 1/48) genes. One *E. coli* isolate originating from the fecal sample was found to harbor both *mcr-4* and *mcr-6* genes and another isolate from the drinking water sample was carrying *mcr-1* and *mcr-8* genes. The majority of the *mcr* positive isolates were categorized under phylogroup A followed by phylogroup B1. The most prevalent sequence typing (ST) was ST1771 (*n* = 4) followed by ST206 (*n* = 3). 100% of the *mcr* positive *E. coli* isolates were multidrug resistant. The most frequently detected virulence genes among *mcr* positive *E. coli* isolates were ast (38%; 18/48) followed by *iss* (23%; 11/48). This is the first research to report the prevalence of *mcr-4, mcr-5, mcr-6, mcr-7,* and *mcr-8* genes in *E. coli* from broiler chickens and farm environments in Malaysia.

**Conclusion:**

Our findings suggest that broiler chickens and broiler farm environments could be reservoirs of colistin-resistant *E. coli*, posing a risk to public health and food safety.

**Supplementary Information:**

The online version contains supplementary material available at 10.1186/s12866-023-03118-y.

## Background

Antimicrobial resistance (AMR) is a major threat to global public health. AMR spreads to the community primarily due to the excessive use of antimicrobials in humans and animals [1]. The use of antimicrobials for disease control or growth promoters in animals causes the commensal microflora to acquire antimicrobial resistance genes (ARGs) through horizontal gene transfer from resistant strains [2]. Evidence shows that AMR in humans can be caused by horizontal transfer of food animal-originated ARGs to human pathogens or through direct transfer of resistant bacteria [3].

*Escherichia coli* (*E. coli*) is an *Enterobacteriaceae* that commonly inhabits the guts of animals and humans. However, it is responsible for many life-threatening infections in humans and animals including chickens. Antimicrobial resistant *E. coli* strains cause a potential risk to public health. Meanwhile, antimicrobial resistant *E. coli* may function as carriers for antimicrobial resistance determinants to its other strain or other bacteria species [4, 5]. Though colistin was previously avoided from human medicine due to its systemic toxicity, its use has been revived due to its efficacy in the treatment of multi-drug resistant (MDR) Gram-negative bacteria [6]. According to World Health Organization (WHO), colistin serves as a last-resort antibiotic that is critically important to human medicine [7]. Colistin has used for a long time in veterinary medicine for disease control and as a growth promoter in food-producing animals [8]. This excessive use of colistin in animals causes antibiotic resistance in bacteria from animals, leading to the emergence of colistin-resistant bacteria which spreads to humans [9]. Resistant microorganisms in humans could have originated from livestock and food producing animals. Colistin-resistant in bacteria was considered as the result of chromosomal mutation until the discovery of the transferable plasmid-mediated gene (*mcr-1*) in 2015 [10]. The emergence of *mcr* related colistin-resistance is a major threat to the treatment of infections. Following the first report of *mcr-1* from China, many studies reported continuously the novel *mcr* genes in *Salmonella* and *E. coli* [11–14].

In addition, following the invention of *mcr-1* in China, an increased rate of colistin-resistant bacteria was reported in food animals including poultry worldwide, especially in Asia [15, 16]. Many countries including Malaysia, have banned the use of colistin in food additives as a growth promoter due to increased colistin-resistant strains in animals [17]. Even though colistin-resistance has reduced after the complete ban of colistin in animal production, a significant colistin-resistance is still being reported from food animals mainly from pigs and poultry throughout the world [17]. To date, the common reported colistin-resistance encoding genes are *mcr*-*1* through *mcr*-10 [18]. Previous studies from Malaysia specifically in Kelantan showed that chicken meat was contaminated with colistin *mcr*-1 encoded resistant *E. coli* [19, 20].

*Escherichia coli* strains are classified into phylogroups of A, B1, B2, C, D, E, F, and clade I/II [21]. Phylogroups B2 and D are associated with virulent extraintestinal pathogenic *E. coli* (ExPEC), whereas phylogroups A and B1 contain mainly commensal *E. coli* strains [22, 23]. Multilocus sequence typing (MLST) is important for understanding the molecular evolution and phylogenetic relationship of important bacteria such as *E. coli* [24]. It helps to detect the emerging *E. coli* sequence type lineages, which are important in the control of AMR in humans and animals. Commensal and environmental bacteria may serve as a reservoir of ARGs that may be transferred to pathogenic bacteria in farm environments. AMR bacteria may be shed with animal feces and contaminate the farm environment. In previous studies, high rates of AMR gene were reported from broiler chicken litter and sewage [25, 26]. Therefore, the purpose of this study was to identify the molecular characteristics of colistin-resistant *E. coli* in broiler chickens and farm environments, which is important for understanding the potential reservoir of ARGs in chicken farms in Kota Bharu and nearby located commercial farms.

## Methods and materials

### Sample collection

Sample size was calculated using the single population formula based on the previous prevalence 52.1% [19]. A total of 453 samples (210 cloacal and 243 environmental samples) were collected from six different farms in Kota Bharu, Malaysia since February-November 2021. Environmental samples collected were drinking water (*n* = 27), sewage water (*n* = 14), fresh droppings (feces) (*n* = 55), feed (*n* = 32), litter (*n* = 20) and environmental swabs (a swab from utilities used in the farm) (*n* = 95). Cloacal and environmental swab samples were collected using Amies transport medium. The water samples were collected using a clean and sterile container. The collected samples were transported to the laboratory in an ice box with an ice pack and the samples were processed within 6 h of sample collection.

$${\varvec{n}}={{\varvec{Z}}}^{2}({\varvec{p}})(1-{\varvec{p}})/{{\varvec{d}}}^{2},$$

Z = Z value (95% CI, z = 1.96)

***p*** = estimated prevalence, ***p*** = 0.521 for previous prevalence [19] of colistin resistant

***d*** = margin of error (0.05)

${\varvec{n}}$ = (1.96)^2^ × 0.521 x(1–0.521)/0.05 = 383, by adding 10% contingency, the sample size (${\varvec{n}}$)=421

### Isolation and identification of *E. coli*

Collected samples were enriched in Buffered Peptone Water (Oxoid, Manchester, UK) and incubated at 37 °C for 24 h. Using sterile wire loop, the enriched bacteria were inoculated to MacConkey agar (Oxoid, Manchester, UK). Lactose fermenter colonies were streaked with Eosin Methylene Blue (EMB) (Oxoid, Manchester, UK) agar and incubated at 37 °C for 24 h. The green metallic sheen colonies were presumptively identified as *E. coli* and the colonies were further tested for biochemical tests such as triple sugar iron agar (TSI), citrate, urea, indole, methyl red and motility. *E. coli* ATCC^®^ 25922 was used as a positive control strain.

### PCR confirmation of isolated *E. coli*

Genomic DNA was extracted using the boiling method as described previously [27]. Extracted DNA of isolated *E. coli* were amplified with species-specific Pho A and E coli primers for further PCR confirmation as used in previous studies [19, 28–31]. The PCR protocol used for the Pho and E coli primers was as previously described [29, 31]. DNA template extracted from *E. coli* ATCC^®^ 25922 strain was used as a positive control and a PCR tube added nuclease free water instead of the DNA template was used as the negative control. In all PCR reactions in this study, PCR products were analyzed using agarose gel electrophoresis and gel images were analysed using GelDoc^©^ Gel Documentation System (Bio-Rad, USA).

### Antimicrobial susceptibility test

Antimicrobial susceptibility testing (AST) of isolated *E. coli* were conducted using Kirby-Bauer disk diffusion method on Mueller-Hinton agar (MHA) (Oxoid, Manchester, UK). The antimicrobial resistant profile of the isolates were determined against 16 antibiotic discs including aztreonam (30 µg), cefotaxime (30 µg), amoxicillin-clavulanic acid (30 µg), ceftazidime (30 µg), ceftriaxone (30 µg), trimethoprim-sulfamethoxazole (25 µg), chloramphenicol (30 µg), tetracycline (30 µg), imipenem (10 µg), meropenem (10 µg), ciprofloxacin (5 µg), ampicillin (10 µg), streptomycin (10 µg), nalidixic acid (30 µg), cefuroxime (30 µg), and gentamicin (10 µg). All the antibiotics were from Oxoid, UK. The zone of inhibition was interpreted based on CLSI guideline. *E. coli* ATCC^®^ 25922 was used as a control strain [32].

### Colistin minimum inhibitory concentration (MIC)

According to the CLSI recommendation, colistin minimum inhibitory concentration (MIC) was determined by broth microdilution (BMD) elusion using Cation-Adjusted Mueller Hinton Broth (CAMHB) [32]. Briefly, four tubes with 10 ml each of CAMHB and colistin discs were thawed to room temperature. Then aseptically one colistin disc (10 µg) was added to the tube labelled as “1 μg/ml”, two colistin discs to the tube labelled as “2 μg/ml” and 4 colistin discs to the tube labeled “4 μg/ml” and no colistin disc was added to the fourth growth control tube. The tubes were vortexed to precipitate the colistin disc into the broth and elute the colistin from the discs by leaving the mix for at 30 min at room temperature. Then after 3–5 freshly grown colonies were transferred to 4–5 ml of sterile saline, the turbidity of bacterial suspension was adjusted to be equivalent to 0.5 McFarland standard. 50 μl bacterial suspension with an approximate inoculum concentration of 7.5 × 10^5^ CFU/ml was added to each of the four tubes [32]. The minimum inhibitory concentration (MIC) was read as the lowest concentration that inhibits the growth of *E coli* isolates after incubating for 16–20 h at 35 °C. The isolates were considered resistant with MIC ≥ 4 μg/ml and intermediate for MIC ≤ 2 μg/ml based on CLSI guideline [32]. *E. coli* ATCC^®^ 25922 was used as a negative control strain.

### Molecular detection of colistin resistance encoding genes

The confirmed *E. coli* isolates were screened for *mcr-1* to *mcr*-9 genes using two separate multiplex PCR. The first multiplex PCR was *mcr*1-5 following previous protocol [33], and the second multiplex PCR was *mcr*6-9 as described previously [34]. PCR products were analyzed using agarose gel electrophoresis and gel images were analysed using GelDoc^©^ Gel Documentation System (Bio-Rad, USA). Selected samples with *mcr* positive *E. coli* were further confirmed by sequencing. All the primers used in this study are summarized in Table 1.

**Table 1:** Forward and reverse primers sequences used in this study

**Primer name**	**Gene name**	**Sequence**	**Amplicon size (bp)**	**Annealing Temperature(**°C)	**Reference**
Pho A-f	*pho*	F: 5′- GTGACAAAAGCCACACCATAAATGCCT-3′	903	56	[29]
Pho A-r		R: 3′-TACACTGTCATTACGTTGCGGATTTGGCGT-5′			
Ecoli-f	*Ecoli*	F: 5′-TGACGTTACCCGCAGAAGAA-3′	832	55	[31]
Ecoli-r		R: 3′-CTCCAATCCGGACTACGACG-5′			
mcr-1_mp_f	*mcr-1*	F: 5′- AGTCCGTTTGTTCTTGTGGC-3′	320	58	[33]
mcr-1_mp_r		R: 3′- AGATCCTTGGTCTCGGCTTG-5′			
mcr-2_mp_f	*mcr-2*	F: 5′- CAAGTGTGTTGGTCGCAGTT-3′	715		
mcr-2_mp_r		R: 3′- TCTAGCCCGACAAGCATACC-5′			
mcr-3_mp_f	*mcr-3*	F: 5′- AAATAAAAATTGTTCCGCTTATG-3′	929		
mcr-3_mp_r		R:3′- AATGGAGATCCCCGTTTTT-5′			
mcr-4_mp_f	*mcr-*4	F:5′-TCACTTTCATCACTGCGTTG-3′	1116		
mcr-4_mp_r		R:3′-TTGGTCCATGACTACCAATG-5′			
mcr-5_mp_f	*mcr-*5	F:5′-ATGCGGTTGTCTGCATTTATC-3′	1644		
mcr-5_mp_r		R:3′-TCATTGTGGTTGTCCTTTTCTG-5′			
mcr-6_mp_f	*mc*r-6	F:5′-AGCTATGTCAATCCCGTGAT-3′	252	55	[34]
mcr-6_mp_r		R:3′-ATTGGCTAGGTTGTCAATC-5′			
mcr-7_mp_f	*mcr*-7	F:5′-GCCCTTCTTTTCGTTGTT-3′	551		
mcr-7_mp_r		R:3′-GGTTGGTCTCTTTCTCGT-5′			
mcr-8_mp_f	*mcr*-8	F:5′-TCAACAATTCTACAAAGCGTG-3′	856		
mcr-8_mp_r		R:3′-AATGCTGCGCGAATGAAG-5′			
mcr-9_mp_f	*mcr*-9	F:5′-TTCCCTTTGTTCTGGTTG-3′	1011		
mcr-9_mp_r		R:3′-GCAGGTAATAAGTCGGTC-5′			
ChuA. 1b	*chu A*	F:5′-TGCCATCAACACAGTATATCC-3′	288	59	[35]
ChuA.2		R:3′-TCAGGTCGCGAGTGACGGC-5′			
YjaA.1b	*yja A*	F:5′-ATCACATAGGATTCTGCCG-3′	211		
YjaA.2b		R:3′-CAGCGGAGTATAGATGCCA-5′			
TspE4C2.1b	*TspE4.C2*	F:5′-AAGGATTCGCTGTTACCGGAC-3′	152		
TspE4C2.2b		R:3′-AACTCCTGATACAGGTGGC-5′			
AceK.f	*arpA*	F:5′-TGATATCACGCAGTCAGTAGC-3′	400		
ArpA1.r		R:3′-CCGGCCATATTCACATAA-5′			
trpAgpC.1	*trpAgpC*	F:5′-ACAAAAAGTTCTATCGCTTCC-3′	219	62	
trpAgpC.2		R:3′-CCTGATCCAGATGATGCTC-5′			
ArpAgpE.f	*ArpAgpE*	F:5′-ACTATTCTCTGCAGGAAGTC -3′	301	59	
ArpAgpE.r		R:3′-CTTCCGATGTTCTGAACGT-5′			
trpBA.f	*trpBA*	F:5′-TCCTGGGACATAATGGTCAG-3′	489		
trpBA.r		R:3′-GTGTCAGAACGGAATTGT-5′			

### Multilocus sequence typing (MLST)

*Escherichia. coli* isolates that were positive for colistin-resistant encoding *mcr* gene were selected for MLST analysis. MLST was performed by sequencing seven housekeeping genes, *adk, fumC, gyrB, icd, mdh, purA*, and *recA*, as described online in, https://enterobase.readthedocs.io/en/latest/mlst/mlst-legacy-info-ecoli.html.

The primers and PCR protocol used are available on the website [36]. The amplified PCR products were sent to Apical 1st base Sequencing service (Apical, Malaysia), to perform a sequence analysis. The alleles and sequence types were assigned from the *E. coli* database at the MLST website, http://enterobase.warwick.ac.uk/.

### Phylogenetic typing of *E. coli* isolates

Quadruplex PCR was used to classify the isolates into phylogroups A, B1, B2 and D using primers chuA, yjaA, TspE4.C2 and arpA according to the revised protocol of Clermont et al. [35]. The isolates of phylogroup A were separated from phylogroup C by trpAgpC primer which is C-specific primers. Similarly, phylogroup D isolates were differentiated from E using ArpAgpE primer and phylogroup F was separated from phylogroup D in the quadruplex PCR as F does not contain *ArpA* gene.

### PCR detection of virulence genes of *E. coli* isolates

The *mcr* positive *E. coli* isolates were assessed for avian pathogenic *E. coli* (APEC) associated virulence genes. Multiplex PCR protocol used to determine the presence of *papC, iucD, irp2, tsh, vat, astA, iss* and *cva/cvi* virulence genes associated with virulence factors as previously described [37].

## Results

**Fig. 1:**
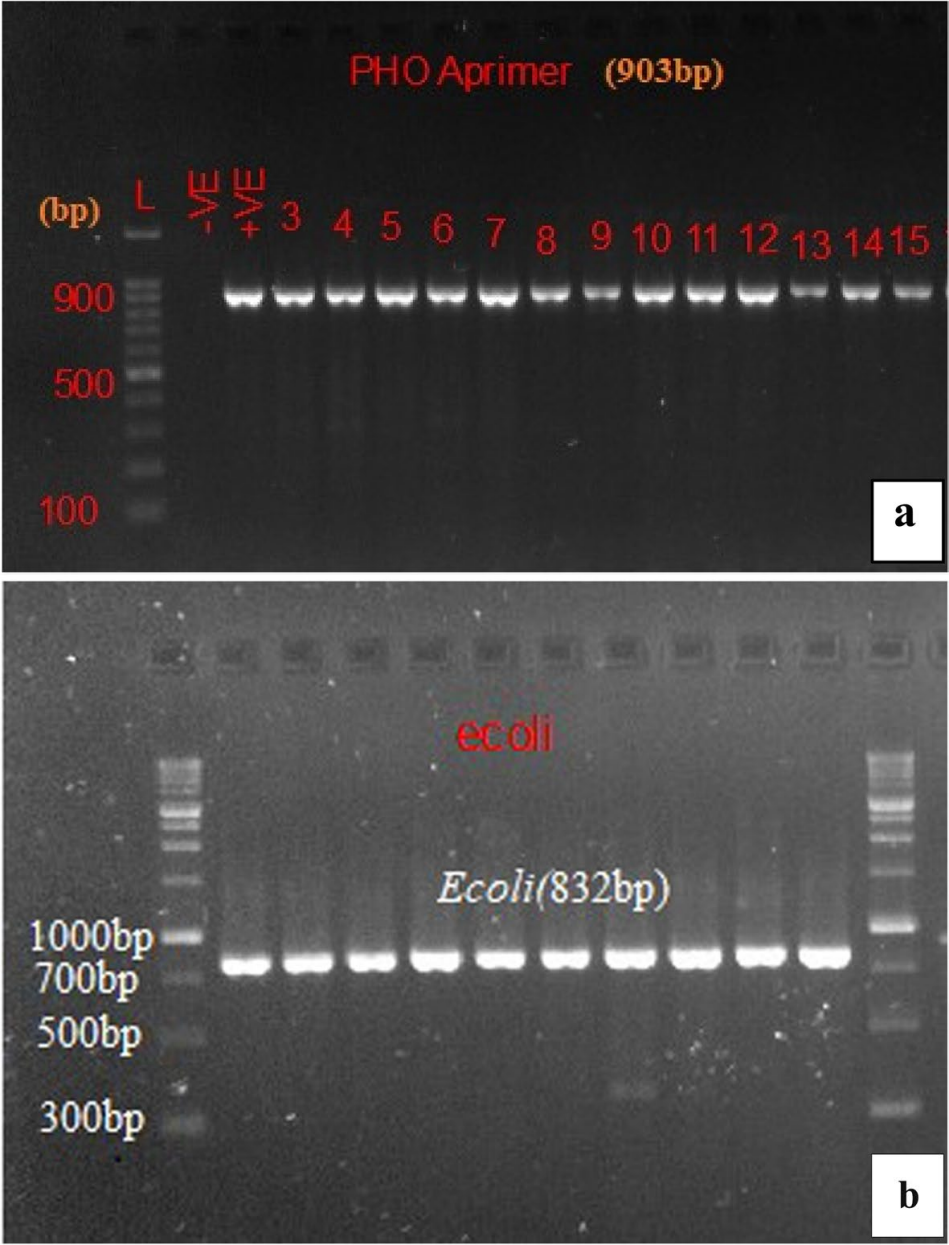
Cropped gel electrophoresis image of amplified PCR product of *Pho A* (**a**) and *E. coli* (**b**) genes

A total of 425(94%) *E. coli* were isolated from the 453 collected samples. Out of these, 203(97%) of *E. coli* strains were isolated from cloacal swab, 83(87%) from environmental swab, 23(85%) from drinking water, 55(100%) from fecal, 31(97%) from feed and 20(100%) from litter. All the isolated *E. coli* were confirmed targeting species specific *pho* and *E. coli* genes (Fig. 1).

### Antimicrobial susceptibility profile

**Fig. 2:**
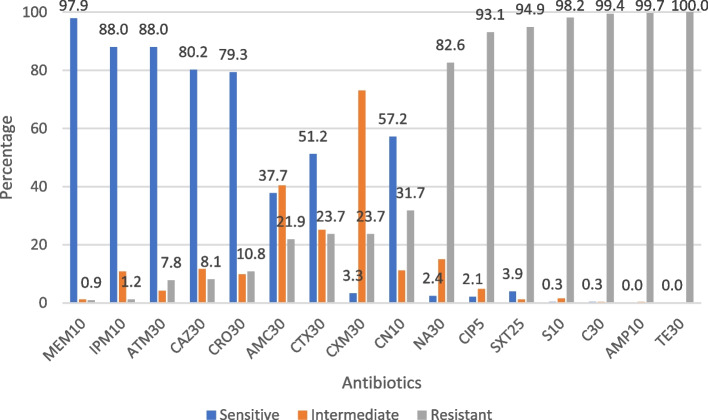
Antimicrobial resistance profiles of *E. coli* isolates from broiler chicken and farm environment in Kelantan, Malaysia *n* = 334

**Fig. 3:**
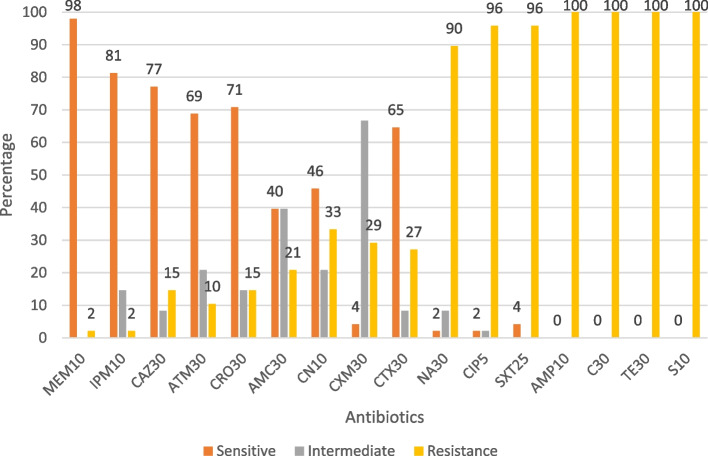
Antimicrobial resistance profiles of *mcr* positive *E. coli* isolates from broiler chicken and farm environment in Kelantan, Malaysia *n* = 48

Isolated *E. coli* was evaluated for antimicrobial susceptibility towards 16 antibiotics of 11 different classes (Fig. 2). All the *mcr* positive *E. coli* isolates were resistant to at least three of the tested antibiotic discs belonging to different classes. The result shows that 100% of the *mcr* positive *E. coli* isolates were resistant to tetracycline, streptomycin, chloramphenicol, and ampicillin (Fig. 3). It was further revealed that 98% of the *mcr* positive *E. coli* strains were susceptible to meropenem. Moreover, compared with *mcr* negative *E. coli*, *mcr* harboring *E. coli* isolates showed higher resistance rates to nalidixic acid, ciprofloxacin, trimethoprim/sulfamethoxazole, and cefotaxime (Fig. 3). Out of 48 *mcr* gene positive *E. coli* isolates 26 (54.2%) of them were with colistin MIC of ≥ 4 μg/ml while the rest, 22 (45.8%) of the *mcr* gene positive isolates were MIC ≤ 2 μg/ml (Table 4).

### Colistin resistance encoding genes

**Fig. 4:**
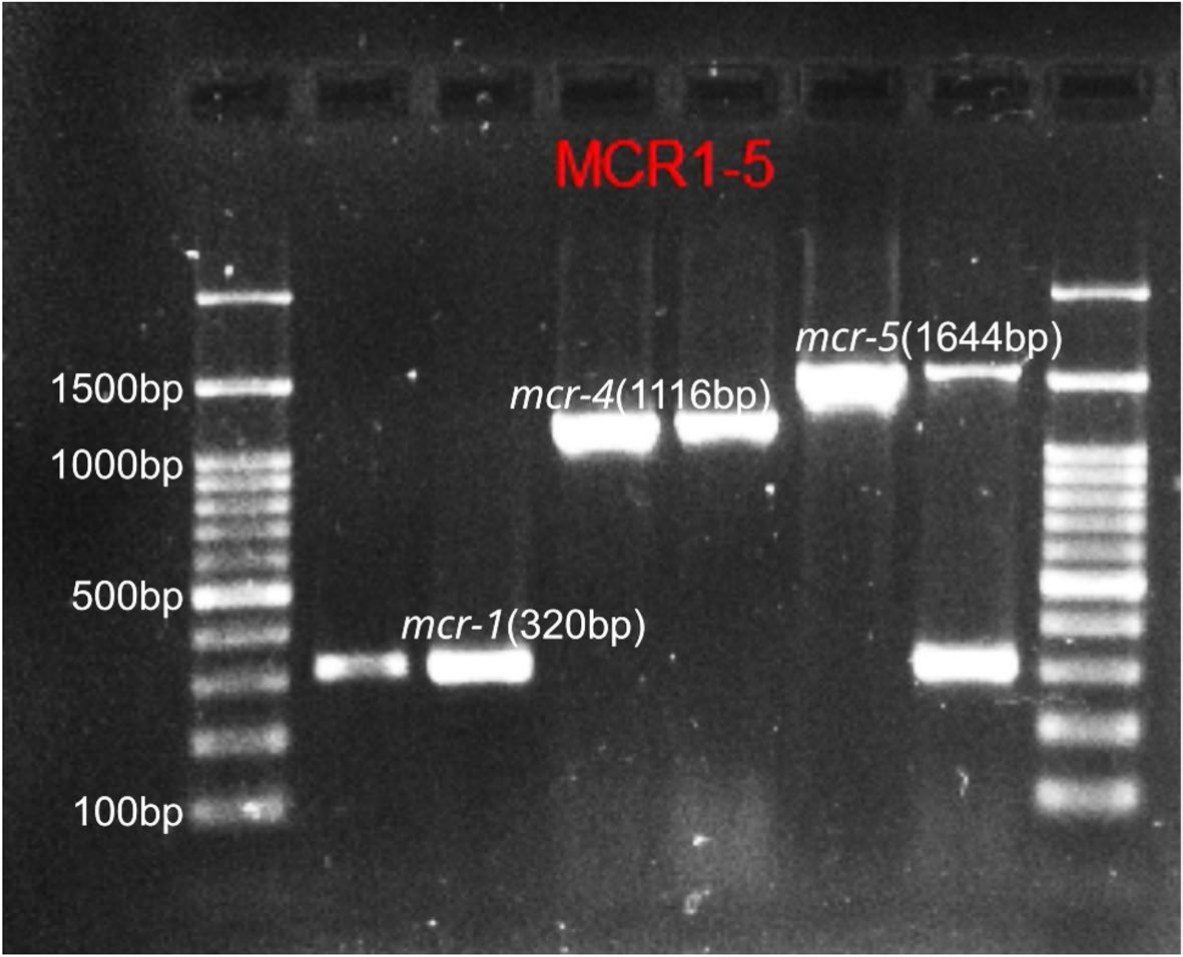
Cropped gel electrophoresis image of amplified PCR product with *mcr-1*, *mcr-4*, and *mcr-5* genes

**Fig. 5:**
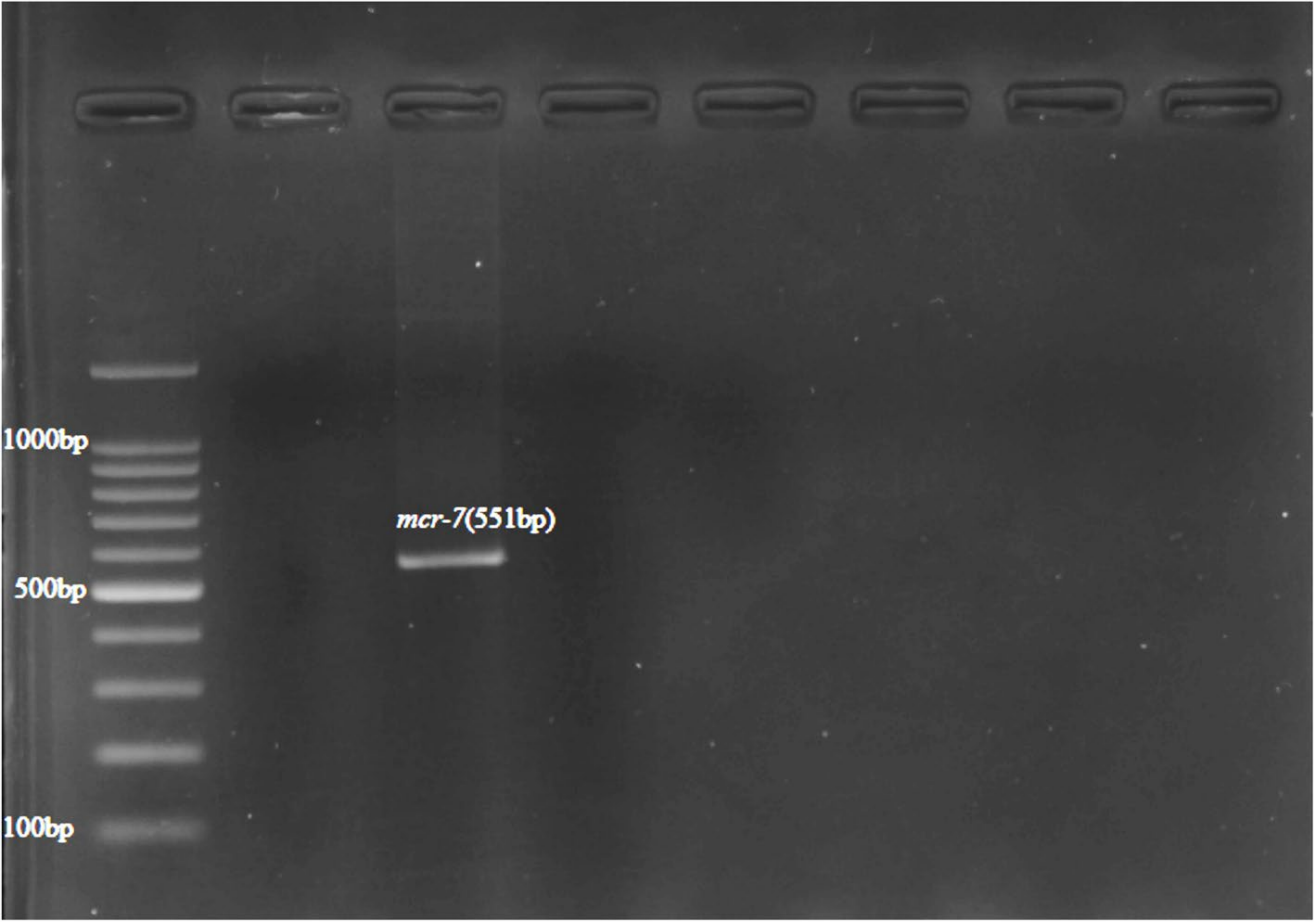
Cropped gel electrophoresis image of amplified PCR product *mcr-7* gene

**Fig. 6:**
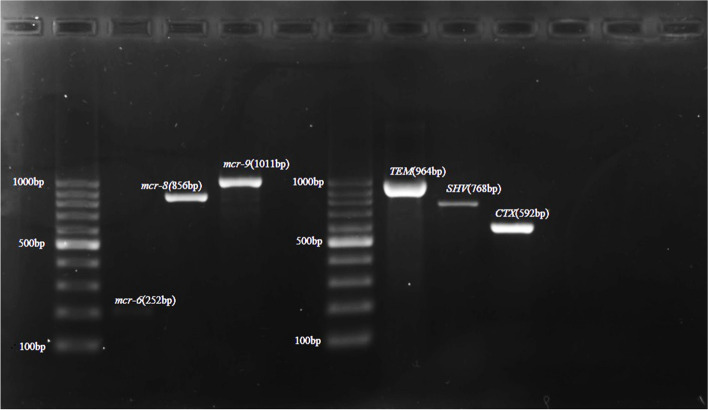
Cropped gel electrophoresis image of amplified PCR product with *mcr-6*, *mcr-8*, and *mcr-9*, and ESBL genes, *TEM*, *SHV* and *CTX* genes

**Fig. 7:**
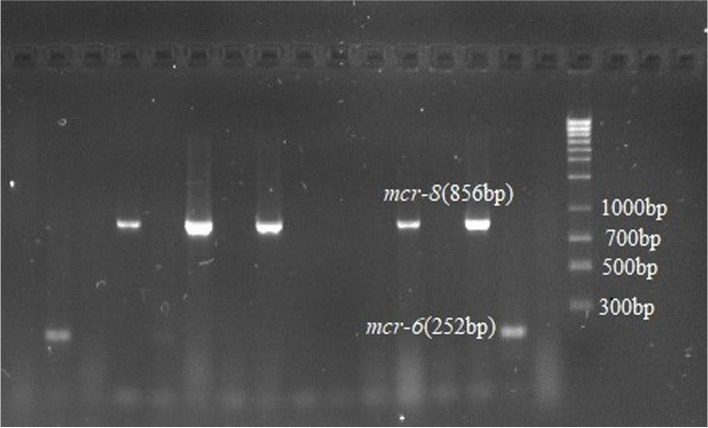
Cropped gel electrophoresis image of amplified PCR product with *mcr-6* and *mcr-8* genes

Colistin-resistance encoding genes were detected using multiplex PCR. Out of the PCR confirmed *E. coli*, 48 (10.8%) isolates were found harboring at least one *mcr* gene. The most prominent *mcr* gene detected was *mcr-1* (47.9%; 23/48), followed by *mcr-8* (18.8%; 9/48), *mcr-7* (14.5%; 7/48), *mcr-6* (12.5%; 6/48), *mcr-4* (2.1%; 1/48), *mcr-5* (2.1%; 1/48), and *mcr-9* (2.1%; 1/48) genes (Table 2). Four (8.3%) isolates harbored more than one gene, *mcr*-4 and *mcr-6*, *mcr-1,* and *mcr*-*8*, *mcr*-*1*and *mcr*-*7* and the fourth one was harboring *mcr-1*and *mcr-5*. In this study out of the *mcr* positive isolates the dominant *mcr* gene detected were *mcr-1*. Majority of the *mcr-1* gene positive *E. coli* were isolated from cloacal and environmental swab samples. Meanwhile, the *mcr*-*1* positive isolates were also detected in food, fecal, litter and drinking water samples. The *mcr*-*4* and *mcr*-*5* gene positive isolates were detected from freshly passed fecal and food samples respectively. In the current study, 33.3% (16/48) of *mcr* positive *E. coli* were from cloacal samples, 29.2% (14/48) from environmental swab,10.4% (5/48) from drinking water,10.4% (5/48) fecal,10.4% (5/48) feed, and 6.3% (3/48) were from litter. The gel electrophoresis image of amplified PCR product with *mcr-1, mcr-4,* and *mcr-5* gene (Fig. 4); *mcr-7* gene (Fig. 5); *mcr-6,mcr-8,* and *mcr-9,* and *ESBL* (*TEM*,* SHV*, and *CTX*) genes (Fig. 6); *mcr-6* and *mcr-8* genes (Fig. 7) are described below.

**Table 2:** Colistin resistance encoding *mcr* genes in *E. coli* isolates from broiler chicken and farm environment in Kelantan, Malaysia (*n* = 48)

**Sample type**	***mcr-1***	***mcr-4***	***mcr-5***	***mcr-6***	***mcr-7***	***mcr-8***	***mcr-9***	**Grand total**
Cloacal Swab	7	-	-	4	1	3	1	16
Drinking water	2	-	-			3	-	5
Environmental	7	-	-	1	4	2	-	14
Fecal	2	1		1	1	-	-	5
Food	3	-	1		1			5
Litter	2	-	-			1		3
Grand Total	23	1	1	6	7	9	1	48

### Phylogenetic typing of *E. coli* isolates

**Fig. 8:**
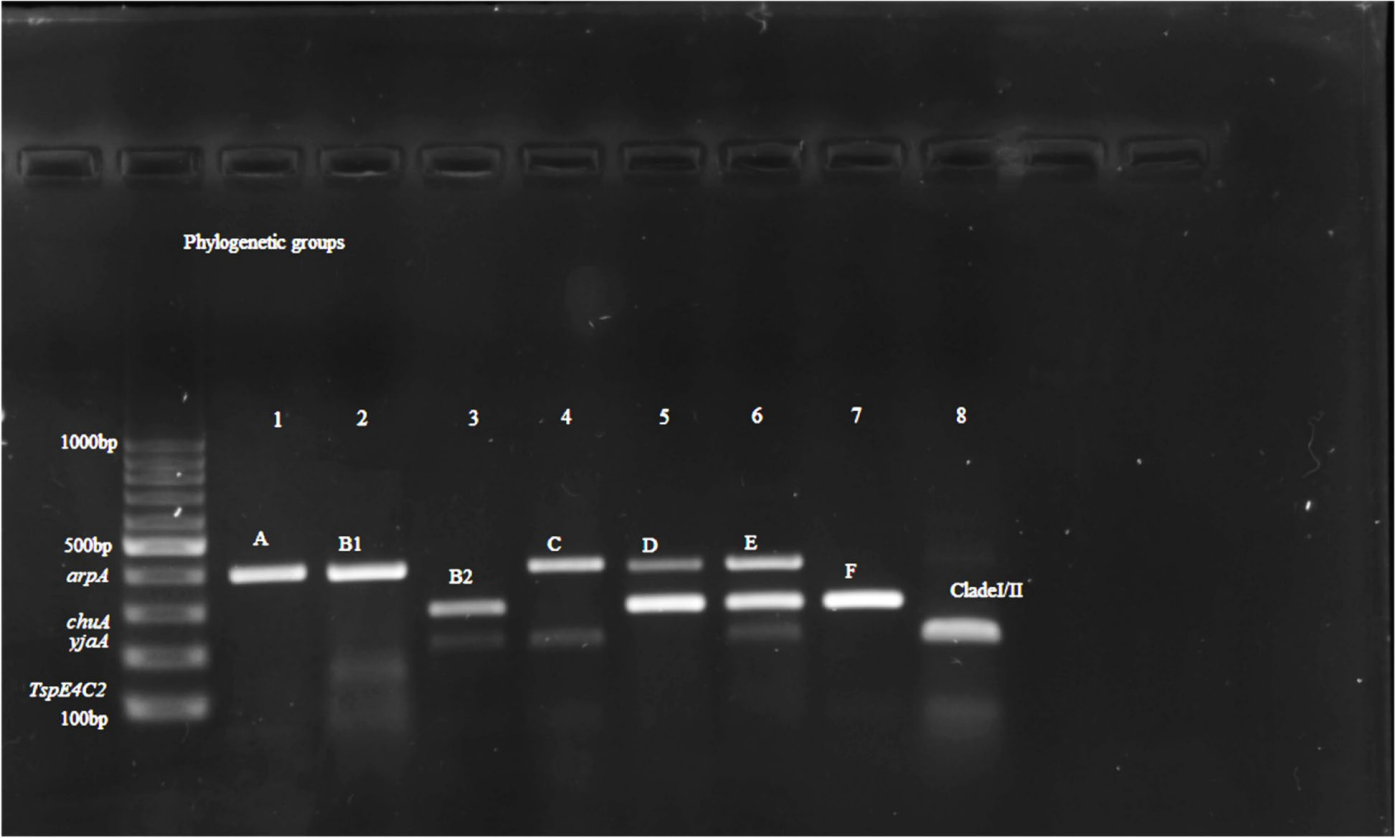
Agarose gel electrophoresis image of *mcr* positive *E. coli* phylogenetic typing. Amplified PCR products with *E. coli* phylogrouping genes; *arpA* (400 bp), *chuA* (288 bp), *yja*A (211 bp) and *TspE4C2* (152 bp); lane 1, + - - -, belonging to phylogroup A; lane 2, + - - + , belonging to group B1; lane 3,- + + -, group B2; lane 4, + - + -, group C; lane 5, + + - -, group D; lane 6, + + + -, group E; lane 7, - + - -,group F; lane 8, - - + -, clade I/II

The majority of the *mcr* positive *E. coli* isolates were assigned to phylogroup A, which is 50% (24/48), followed by B1(12.5%) (Table 3). While the rest belonged to phylogroup C (*n* = 5, 10.4%); D (*n* = 5, 10.4%); E (*n* = 2, 4.2%); F (*n* = 2, 4.2%); Clade I or II (*n* = 2, 4.2%) and B2 (*n* = 1, 2.1%). Most isolates with phylogroup A originated from cloacal (*n* = 6) and poultry environment (*n* = 18). Figure 8 below shows the gel electrophoresis image of the four genes used to separate the isolates to phylogenetic groups.

**Table 3:** Phylogroup of isolated *E. coli* isolates from broiler chicken and farm environment in Kelantan, Malaysia *n* = 48

Sample Type	Phylogroup								
	A	B1	B2	C	Clade I/II	D	E	F	Unknown
Cloacal	6	3	1	2	1	2	1	1	
Evt swab^a^	8	1					1	1	1
Fecal	3	-			1				
Food	4	-		2					
Litter	1	-		1		1			
Swage	-					1			
Water	2	2				1			
Total	24	6	1	5	2	5	2	2	1
Percentage (%)	50.0	12.5	2.1	10.4	4.2	10.4	4.2	4.2	2.1

^a^*Evt swab* Environmental swab

### Multilocus sequence typing and virulence genes

Among the forty-eight *mcr* encoding colistin-resistant isolates, 23 *mcr* gene and virulence gene positive isolates were selected for MLST sequencing. Tthe MLST result of the isolates shows that the *E. coli* isolates were widely diverse. The most prevalent STs found were ST1771 (*n* = 4) followed by ST206 (*n* = 3). ST 1771 was found from cloacal, food and drinking water source isolates, ST206 was found from cloacal, environmental swab and drinking water sources. In this study among 18STs, the majority belonging to phylogroup A (44.4%) (Table 4). All the ST1771 except one were phylogroup A, one was group C and two of the ST206 were phylogroup A whereas one was phylogroup C. *E. coli* strains with ST165, ST206, ST1771, ST162, ST398, ST1285 and ST106 were found associated with *mcr*, *CTX* and *SHV* genes originate from fecal, cloacal, environmental swab, water, and litter sources. Moreover, ST165, ST1771, ST155, ST48, ST206, ST162, ST159, ST38 were found positive for *ast, iss, irp2*, or /and *iucD* virulence genes, which belongs to phylogroup A, B1, D, C, E and Clade I/II. The most frequently detected virulence genes among *mcr* positive *E. coli* isolates were *ast* (38%; 18/48) followed by *iss* (23%; 11/48), *irp2* (17%; 8/48), *iucD* (13%; 6/18), *papC*(6%; 3/48) and *tsh* (2%; 1/48) genes.

**Table 4:** Phenotypic and genotypic characterization of *mcr* positive *E. coli* from broiler chicken and farm environment (*n* = 48)

**Isolate ID**	**Source**	**ST**	**Resistant Antibiotics**	**Colistin MIC(µg/ml)**	**Phylogroup group**	***mcr* and ESBL gene**	**Virulence gene**
KBF1	Fecal	165	CIP,AMP,CHL,TET,SXT,STR,CTX,ATM,NAL,CRO,CXM	≥ 4	A	*mcr-4, mcr-6, CTX*	*ast, iss,irp2*
F6(2)	Fecal	605	CIP,AMP,CHL,TET,SXT,STR,NAL,GEN	≥ 4	A	*mcr-1*	*ast,irp2*
19D(2)	Evt swab	7506	CIP,AMP,CHL,TET,SXT,STR,CTX,NAL,CXM,GEN	≤ 2	B1	*mcr-7*	*iss*
BFd8H3	Food	1771	CIP,AMP,CHL,TET,SXT,STR,NAL,	≤ 2	A	*mcr-8*	*ast,iss,irp2*
ZC5B	cloacal	1771	CIP,AMP,CHL,TET,SXT,STR,NAL,	≤ 2	C	*mcr-8*	*ast*
B9H2	cloacal	155	CIP,AMP,CHL,TET,SXT,STR,	≤ 2	E	*mcr-1*	*ast, iss,irp2,iucD*
12B	cloacal	48	AMC,CIP,AMP,CHL,TET,SXT,STR,CTX,NAL,	≤ 2	Clade I or II	*mcr-1*	*ast, iss,irp2,iucD*
3BA	cloacal	1703	CIP,AMP,CHL,TET,SXT,STR,NAL	≤ 2	A	*mcr-1*	*ast,pap C*
ZC4BN	cloacal	206	CIP,AMP,CHL,TET,STR,	≤ 2	C	*mcr-8, SHV*	*ast*
C39H4	cloacal	1771	CIP,AMP,CHL,TET,SXT,STR,NAL	≤ 2	A	*mcr-8, CTX*	*ast,papC*
YC1H2	cloacal	354	CIP,AMP,CHL,TET,SXT,STR,NAL,GEN	≥ 4	F	*mcr-6*	*ast*
Dr7H2	Water	1771	CIP,AMP,CHL,TET,SXT,STR,NAL,	≥ 4	A	*mcr-8*	*irp2*
E15(2)	Evt swab	206	CAZ,AMC, CIP,AMP,CHL,TET,SXT,STR,CTX,NAL,CXM, GEN	≥ 4	A	*mcr-1*	*ast,tsh,iss*
KB4A	cloacal	162	CIP,AMP,CHL,TET,SXT,STR,NAL,GEN	≤ 2	B1	*mcr-9, CTX*	*ast,iss, iuc D*
BFd5H4	Food	159	CIP,AMP,CHL,TET,SXT,STR,NAL,CXM,	≥ 4	C	*mcr-1,mcr-5*	*ast,iss, iuc D*
B8H4	cloacal	11,630	AMC, CIP,AMP,CHL,TET,SXT,STR,CTX,NAL,CXM	≤ 2	B1	*mcr-6*	*ast, iuc D*
B7H4	cloacal	38	CIP,AMP,CHL,TET,SXT,STR,NAL,GEN	≥ 4	D	*mcr-7, mcr-1*	*ast,iss,papC,iucD*
E43A	Evt swab	398	CIP,AMP,CHL,TET,SXT,STR,NAL,	≥ 4	F	*mcr-6, CTX*	*ast,iss*
Drw3	Water	206	CIP,AMP,CHL,TET,SXT,STR,CTX,NAL,CRO,	≥ 4	A	*mcr-1, mcr-8, SHV*	*ast*
Drw7	Water	1285	CIP, AMP,CHL,TET,SXT,STR,NAL,CXM,GEN	≥ 4	B1	*mcr-1, SHV*	*_*
WF5	Swage	1140	CAZ,AMC,CIP,AMP,CHL,TET,SXT,STR,NAL,CXM	≥ 4	D	*mcr-7*	*iss,irp2*
L25B	Litter	106	CAZ,AMC,CIP,AMP,CHL,TET,SXT,STR,CTX,ATM,NAL,CRO,CXM	≥ 4	D	*mcr-1, SHV,CTX*	*irp2*
Food_G	Food	43	AMP,CHL,TET,SXT,STR,NAL	≥ 4	C	*mcr-1*	*_*
7a	cloacal	_	CIP,IPM,AMP,CHL,TET,SXT,STR,NAL	≥ 4	D	*mcr-6*	_
B2H1	cloacal	_	CIP,AMP,CHL,TET,SXT,STR,NAL,	≥ 4	B2	*mcr-6*	*_*
BFood7H1	Food		CIP,AMP,CHL,TET,SXT,STR,NAL,CXM	≤ 2	A	*mcr-7*	*_*
Drw1	Water	_	CIP,AMP,CHL,TET,SXT,STR,CTX,NAL,	≥ 4	D	*mcr-8*	*_*
Drw4	Water	_	CIP,AMP,CHL,TET,SXT,STR,CTX,NAL,CRO,	≥ 4	B1	*mcr-8*	*_*
E1	Evt swab	_	CIP,AMP,CHL,TET,SXT,STR,CTX,NAL,CXM,GEN	≥ 4	A	*mcr-1*	*_*
E21	Evt swab		CIP,AMP,CHL,TET,SXT,STR,	≤ 2	Unknown	*mcr-7*	
E22	Evt swab		CIP, AMP,CHL,TET,SXT,STR,NAL,	≤ 2	A	*mcr-1*	*_*
E28	Evt swab		CIP,AMP,CHL,TET,SXT,STR,NAL,	≤ 2	E	*mcr-1*	
EVT11	Evt swab		AMC,CIP,AMP,CHL,TET,SXT,STR,NAL,	≤ 2	A	*mcr-7*	
EVT16	Evt swab	_	CIP,AMP,CHL,TET,SXT,STR,NAL,GEN	≥ 4	A	*mcr-1*	*_*
EVT21	Evt swab	_	CIP,AMP,CHL,TET,SXT,STR,ATM,NAL,	≥ 4	A	*mcr-1*	*_*
F1	Fecal	_	CIP,AMP,CHL,TET,SXT,STR,NAL,	≥ 4	A	*mcr-7*	*_*
L16	Litter	_	CIP,AMP,CHL,TET,SXT,STR,NAL,	≥ 4	A	*mcr-1*	*_*
L17	Litter	_	CIP,AMP,CHL,TET,SXT,STR,ATM,NAL	≥ 4	C	*mcr-8*	*_*
YC23H2	cloacal		CIP,AMP,CHL,TET,SXT,STR,	≤ 2	A	*mcr-1*	*ast*
ZC13H1	cloacal		CIP, AMP,CHL,TET,SXT,STR,NAL,GEN	≤ 2	A	*mcr-1*	
ZC22H1	cloacal	_	AMP, CHL,TET,STR	≥ 4	B1	*mcr-1*	*_*
EVT13	Evt swab		AMC, CIP,AMP,CHL,TET,SXT,STR,NAL,CXM	≤ 2	A	*mcr-1*	
Fecal	Fecal		CIP, AMP,CHL,TET,SXT,STR,CTX,NAL,CRO,CXM	≤ 2	Clade I or II	*mcr-1*	
FoodB	Food	_	CAZ, MEM,CIP,AMP,CHL,TET,SXT,STR,CTX,ATM,NAL,CRO,CXM	≥ 4	A	*mcr-1*	*_*
FoodD	Food		AMC, CIP,AMP,CHL,TET,SXT,STR,CTX,ATM,NAL,CRO,CXM,GEN	≤ 2	A	*mcr-1*	
11a	cloacal		AMC, CIP,AMP,CHL,TET,SXT,STR,CTX,NAL,CXM	≤ 2	A	*mcr-6*	
E2	Evt swab	_	CIP, AMP, CHL,TET,SXT,STR,NAL,GEN	≥ 4	A	*mcr-8*	_
12a	cloacal		CAZ,CIP,AMP,CHL,TET,SXT,STR,CTX,ATM,NAL,CRO,CXM,GEN	≤ 2	A	*mcr-6*	

*ATM* Aztreonam, *CTX* Cefotaxime, *AMC* Amoxicillin-clavulanic acid, *CAZ* Ceftazidime, *CRO* Ceftriaxone, *SXT* Trimethoprim-sulfamethoxazole, *CHL* Chloramphenicol, *TET* Tetracycline, *IMP* Imipenem, *MEM* Meropenem, *CIP* Ciprofloxacin, *AMP* Ampicillin, *STR* Streptomycin, *NAL* Nalidixic acid, *CXM* Cefuroxime, *GEN* Gentamicin

## Discussion

In the present study, 425 (94%) *E. coli* were isolated from 453 cloacal and environmental samples. Forty-eight (10.8%) of *E. coli* isolates were positive for at least one of the colistin-resistance encoding *mcr* gene. 100% of the *mcr* positive isolates were multidrug-resistant, which have the potential to disseminate to human and farm animals.

In this study 100% of the *mcr* positive *E. coli* isolates were resistant to tetracycline, streptomycin, chloramphenicol, and ampicillin. This correlation between *mcr* and resistance of these antibiotics might be due to the coexistence of *mcr* and ESBLs in the plasmids that could also harbor resistant genes of different classes of antimicrobials and have the trait of being multi-drug resistant [38, 39]. Among the *mcr* positive strains, 98% were susceptible to meropenem, a carbapenem antibiotic. Moreover, *mcr* harboring *E. coli* isolates exhibited higher resistance rates against nalidixic acid, ciprofloxacin, trimethoprim/sulfamethoxazole, and cefotaxime as compared with *mcr* negative *E. coli*. High resistance rate to tetracycline and ampicillin was also reported in *E. coli* isolates from chicken in Malaysia and Vietnam [20, 40]. The susceptibility test for colistin by disc diffusion and E-test is difficult due to polymyxins’ poor agar diffusion [41], and reliable reference break point is not available. Therefore, in this study MIC was used for colistin susceptibility test. This study revealed that 26(54.2%) of the *mcr* positive isolates have MICs of colistin ≥ 4 µg/ml, suggesting that the isolates are genotypically and phenotypically resistant to colistin. However, 22(45.8%) of genetically resistant isolates were phenotypically susceptible to colistin, with MIC ≤ 2 µg/ml. These discrepancies might be due to sensitivity difference between broth micro dilution and PCR detection methods in colistin resistance. A previous study stated that broth micro dilution has a 71.4% sensitivity rate in the detection of *mcr-1* positive *Enterobacteriaceae* [42]. Nonetheless, PCR is widely considered as the gold standard method in detecting colistin-resistance [10, 33].

An interesting finding of this research is that 48 (10.8%) of the *E. coli* isolates were positive for at least one colistin-resistance encoding *mcr* gene. Out of the *mcr* genes *mcr-1* was the prominent gene (47.9%; 23/48). Studies from Malaysia have found 23.08% *mcr-1* encoding *E. coli* from broiler chicken, while 52.1% of *E. coli* from chicken meat was carrying *mcr*-*1* gene [19, 20, 43]. Similarly, 23.08% *mcr-1* harboring *Klebsiella pneumonia* strains were reported from pigs in Malaysia [44]. Furthermore, *mcr-1* gene positive *Enterobacteriaceae* isolates have been found in food animals, humans, and environment globally, with high prevalence in food animals compared to humans [15, 45–47]. This widespread and relatively increased prevalence of *mcr-1* gene related colistin-resistance in farm animals and retail meat indicate that food animals could be a potential reservoir for human transmission [48]. Recently, following the ban of colistin use as food additive in food animals in many countries, the prevalence of *mcr-1* gene positive bacteria in food animals, including chickens has decreased [49].

Moreover, we found other *mcr* genes including *mcr*-*4* (2.1%; 1/48), *mcr*-*5* (2.1%; 1/48), *mcr*-*6* (12.5%; 6/48), *mcr-7* (14.5%; 7/48), *mcr-8* (18.8%; 9/48) and *mcr-9* (2.1%; 1/48) for the first time in Malaysia. However, none of the isolates of this research were positive for *mcr-2* and *mcr-3* genes. A study from China were reported *mcr-4* and *mcr-5* from chicken origin isolates [50]. In addition, the *mcr-5* gene was also detected from chicken origin from Singapore, Brazil, and Paraguay [51, 52]. The *mcr-*5 and *mcr*-*9* genes were found harbored in *E. coli* from chickens in Brazil [53]. In addition, *mcr-9* genes were detected in *Salmonella* isolated from chicken meat in Korea and from USA in *Salmonella* and *E. coli,* even though the bacteria were not associated with colistin resistance [54, 55]. Meanwhile, *mcr*-*7* and *mcr-8* genes were detected from *Klebsiella pneumonia* from chicken and animal origin respectively in China [14, 56]. The *mcr-6* was revealed from *Moraxella* species of pig origin from Britain [57]. This indicates that *mcr* genes are widely disseminated among different bacterial species and have spread globally. A review showed that among the colistin-resistance *mcr* genes, *mcr-1* and *mcr-9* have become globally spread [58]. In our research, four (8.3%) *mcr* positive *E. coli* isolates were harboring more than one *mcr* genes. One isolate tested positive for both *mcr-4* and *mcr-6*, while another carried *mcr-1* and *mcr-8*. The third harbored *mcr-1* and *mcr-7* and the fourth *E. coli* isolate was positive for both *mcr-1* and *mcr-5*. The first two isolates belonged to phylogroup A, while the latter belonged to phylogroup D. These strains were isolated from chicken fecal samples, drinking water and cloacal swab. Majority of the *mcr-1* gene positive *E. coli* were detected from cloacal and environmental swab samples. Meanwhile, *mcr-1* gene positive *E. coli* were also detected in food, feces, litter and drinking water samples. The majority of the *mcr* genes positive *E. coli* were detected from chicken farm environment samples. Similarly, *mcr-1* encoding* E. coli* strains were reported from litter, feed and drinking water in study from Lebanon and Indonesia [59, 60]. Shedding of the AMR bacteria and determinants from the feces into the farm environment causes dissemination of AMR to the chicken and animal handlers. As the litter often be used as a fertilizer, it also serve as a reservoir of resistant bacteria and determinants to agriculture and the environment [61]. Few previous studies in Asia showed *mcr* genes ranging from 10.5%-36.6% of *mcr-1* in Bangladesh, Indonesia chicken [46, 60, 62]. To best of our knowledge*, mcr-4, mcr-5, mcr-6, mcr-7, mcr-8*, and *mcr-9* genes were not reported from broiler chicken and farm environment origin in Malaysia.

Most of the isolates in our study were assigned to phylogroup A followed by B1, which is consistent with previous studies based on healthy broilers and environment [20, 63, 64]. In addition, phylogroup D (10.4%), F (4.2%) and B2 (2.1%) were found in the current study. Several studies have shown phylogroups B2 and F from APEC colibacillosis-causing strains that were a causative agent of human ExPEC [63, 65–67]. Phylogenetic group B2 and D are the most virulent causes of ExPEC infections in humans and chickens in France and China [22, 68].

In the present study, *E. coli* ST155, ST48 and ST38 harbored four virulence genes, including *ast, iss, irp2* and *iuc*D genes. *E. coli* ST155 and ST48 were caring *mcr-1,*while *E. coli* ST38 were positive for *mcr-1* and *mcr-7*. *E. coli* ST165 were related to *ast, iss* and *irp2* genes which was positive for *mcr-4, mcr-6 and CTX,* while *E. coli* ST206 were carrying *ast, tsh and iss* genes that was positive for *mcr-1*. *E. coli* ST155 isolates were also found in broiler chicken samples from previous research from Malaysia [20, 69]. Previous study reported the presence of *E. coli* ST155, ST48, and ST398 with MDR trait in human and chicken farm environment in Nigeria [70]. Furthermore *E. coli* ST155 was detected from APEC [71, 72] and from both APEC and human ExPEC [73] strains in previous studies. ST206 has been reported in China from human clinical samples, harboring both the *mcr-1* and carbapenems gene *blaNDM*-5 genes [74]. *E. coli* ST206 isolates were also associated with ESBL gene (*CTX-M*-27) from human and animal sources in Nigeria [75]. ST48 was previously reported in systemic *E. coli* associated with APEC in UK [76]. *E. coli* ST38 was isolated from human EXPEC from several studies around the globe [77]. In the present study, *E. coli* ST155, ST48, ST38, ST398 and ST206 were positive for APEC associated virulence genes. Furthermore, these *E. coli* STs were reported as potentially zoonotic human ExpEC. We detected several virulence genes, including *papC, iucD, irp2, tsh, ast* and *iss* genes from the *mcr* positive *E. coli* isolates. The *ast* (38%,18/48) were the most frequently detected virulence gene followed by *iss* (23%,11/48), *irp2* (17%, 8/48), *iucD* (13%, 6/18), *papC*(6%, 3/48) and *tsh* (2%,1/48) genes. The detection of Inspect and ExPEC virulent genes from apparently healthy broilers and farm environment indicates that healthy chickens and farm environment can be the reservoir of *mcr* positive virulent APEC. Similarly, intestinal *E. coli* were found harboring ExPEC including APEC associated genes as reported in previous studies [76, 78, 79]. These virulence genes, including *papC, iucD, irp2, tsh, astA* and *iss* genes has been reported from previous study associated with APEC [80].

## Conclusion and recommendations

This study found that colistin-resistant *E. coli* in broiler chickens and chicken farm environment is high, despite a decrease observed in previous studies following the ban of colistin from animal food additives. However, the *E. coli* isolates harbored a variety of *mcr* genes and were highly resistant to antibiotics such as tetracycline, aminoglycoside, chloramphenicol, penicillin, quinolone, fluoroquinolone, sulfonamide, and cephalosporin. All the *mcr* genes positive *E. coli* isolates displayed resistance against multiple antibiotics. Among *E. coli* isolates from cloacal and farm environments, *mcr-1* was the dominant *mcr* gene. Furthermore*, mcr-4* and *mcr-5* genes were found in faecal and feed samples respectively. This is the first study to report the prevalence of *mcr-4, mcr-5, mcr-6, mcr-7, mcr-8,* and *mcr-9* genes in *E. coli* isolated from Malaysian broiler chickens and farm environments. Our findings suggested that MDR colistin-resistant *E. coli* strains carry virulence genes could be found in broiler chickens and broiler farm environments. These strains pose a high risk of spreading to humans, animals, and the environment. Based on our findings, we recommend stricter regulation of antibiotic use in farm animals since livestock and environments have a vital role in the transmission of antibiotic-resistant bacteria.

### Limitation of the study

This study was limited in scope and did not cover all districts in Kelantan state and other Malaysian states. In addition, samples were collected only from broiler chickens reared under intensive production systems. Diversifying the study by including different poultry production systems including layers, breeders, and backyard poultry farms from representative poultry farms across the country may give a better and more accurate epidemiological information on the resistant bacteria. Moreover, this study did not detect plasmids of the isolated strains.

### Supplementary Information

**Additional file 1: Figure1a.** Original gel electrophoresis image of *Pho A *genes. **Figure 1b.** Original gel electrophoresis image of *Ecoli* genes. **Figure 4.** Original gel electrophoresis image of amplified PCR product with *mcr-1, mcr-4*, and *mcr-5* genes. **Figure 5.** Original gel electrophoresis image of amplified PCR product *mcr-7* gene. **Figure 6.** Original multiplex PCR6-9, gel image of *mcr-6*, *mcr-8*, *mcr-9* and ESBL genes. **Figure 7.** Original gel electrophoresis image of amplified PCR product with *mcr-6* and *mcr-8* genes. **Figure 8.** Agarose gel electrophoresis image of *mcr *positive E. coli phylogenetic typing. Amplified PCR products with E. coli phylogrouping genes; *arpA* (400bp), *chuA* (288bp), *yjaA* (211bp) and *TspE4C2* (152bp); lane 1,+ - - -, belonging to phylogroup A; lane 2,+ - - +, belonging to group B1; lane 3,- + + -, group B2; lane 4,+ - + -, group C; lane 5,+ + - -, group D; lane 6,+ + + -, group E; lane 7,- + - - ,group F; lane 8,- - + -, clade I/II.

## Data Availability

The datasets used and/or analyzed during the current study are available from the corresponding authors on reasonable request.
