# Supplementary material for: Phenotypic and genotypic characterization of colistin-resistant Escherichia Coli with mcr-4, mcr-5, mcr-6, and mcr-9 genes from broiler chicken and farm environment
Source: BMC Microbiol. 2023 Dec 8;23:392. doi: 10.1186/s12866-023-03118-y (PMC10704802; doi:10.1186/s12866-023-03118-y)
Supplement: Supplementary file 1 — Additional file 1: Figure1a. Original gel electrophoresis image of Pho A genes. Figure 1b. Original gel electrophoresis image of Ecoli genes. Figure 4. Original gel electrophoresis image of amplified PCR product with mcr-1, mcr-4, and mcr-5 genes. Figure 5. Original gel electrophoresis image of amplified PCR product mcr-7 gene. Figure 6. Original multiplex PCR6-9, gel image of mcr-6, mcr-8, mcr-9 and ESBL genes. Figure 7. Original gel electrophoresis image of amplified PCR product with mcr-6 and mcr-8 genes. Figure 8. Agarose gel electrophoresis image of mcr positive E. coli phylogenetic typing. Amplified PCR products with E. coli phylogrouping genes; arpA (400bp), chuA (288bp), yjaA (211bp) and TspE4C2 (152bp); lane 1,+ - - -, belonging to phylogroup A; lane 2,+ - - +, belonging to group B1; lane 3,- + + -, group B2; lane 4,+ - + -, group C; lane 5,+ + - -, group D; lane 6,+ + + -, group E; lane 7,- + - - ,group F; lane 8,- - + -, clade I/II. [file 12866_2023_3118_MOESM1_ESM.docx]

**
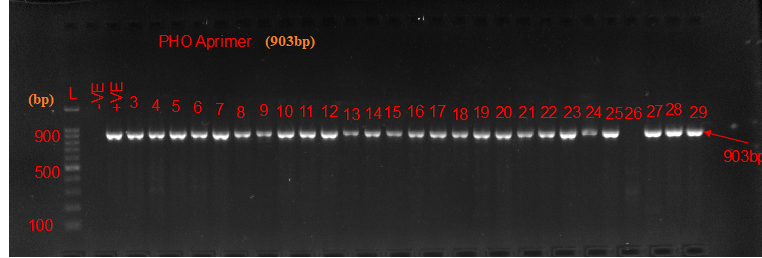
**

**a**

Figure1a. Original gel electrophoresis image of *Pho A* genes

**
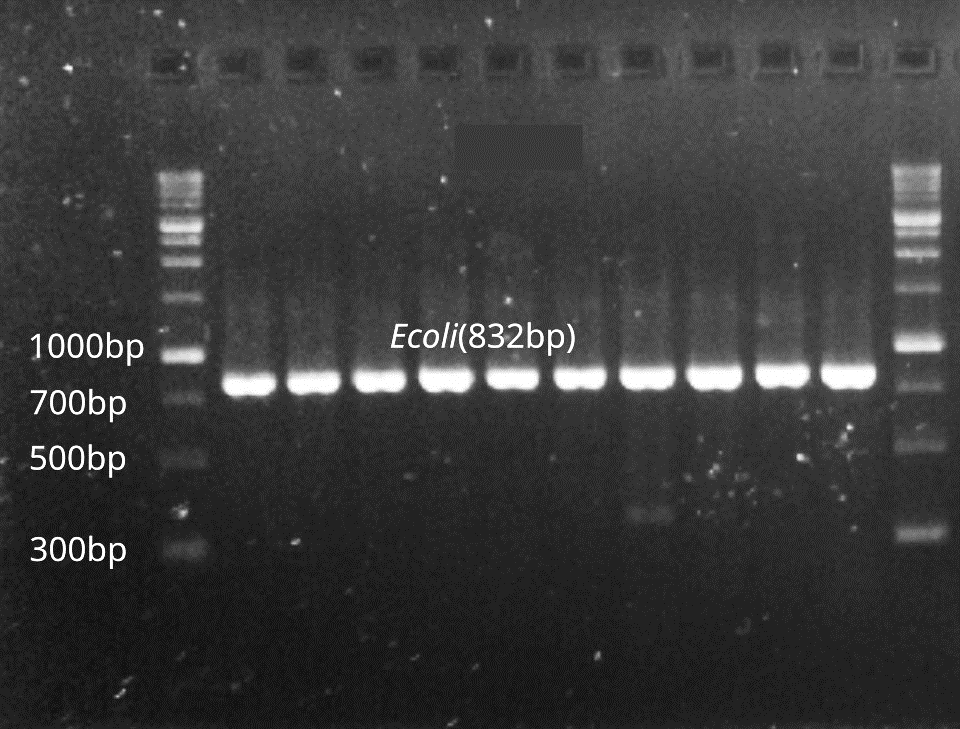
**

**b**

Figure 1b. Original gel electrophoresis image of *Ecoli* genes

**
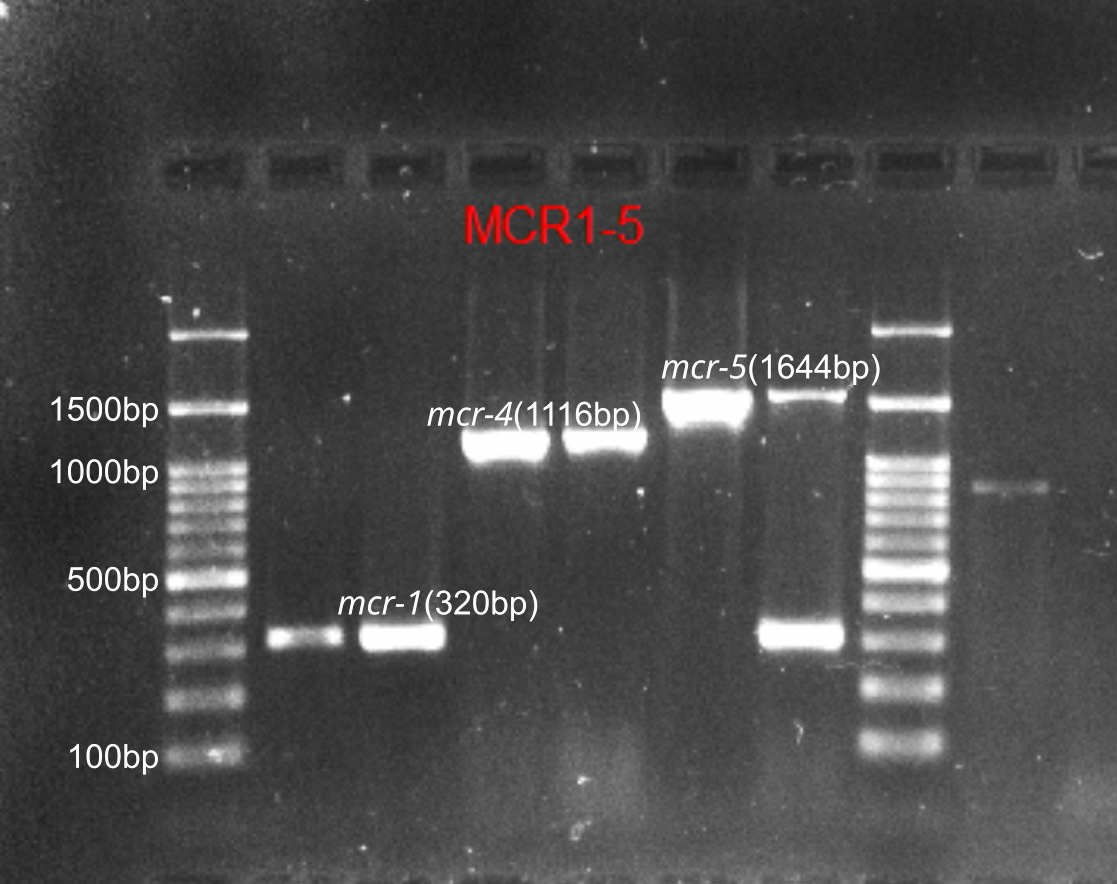
**

Figure 4. Original gel electrophoresis image of amplified PCR product with mcr-1, mcr-4, and mcr-5 genes


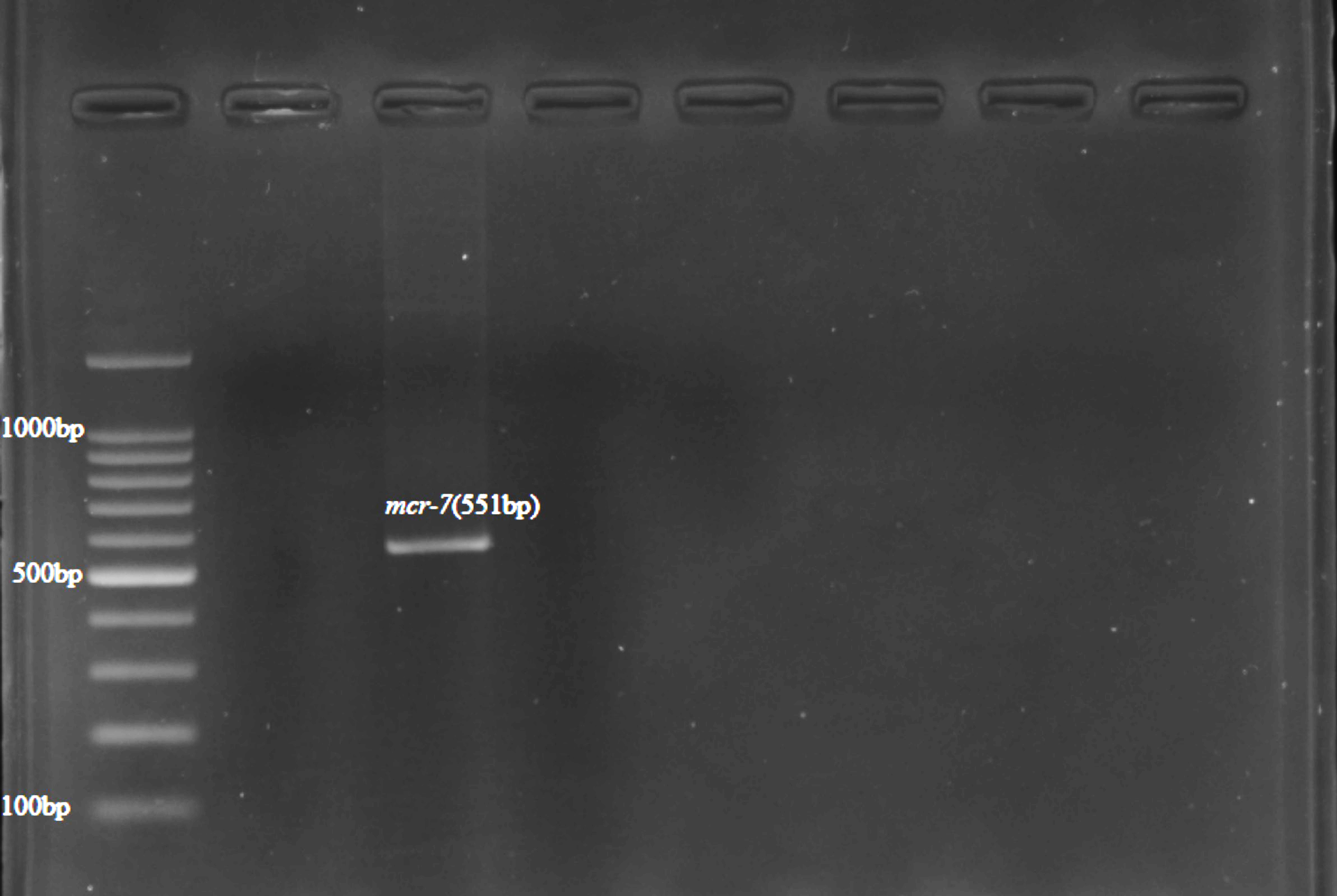


Figure 5. Original gel electrophoresis image of amplified PCR product mcr-7 gene


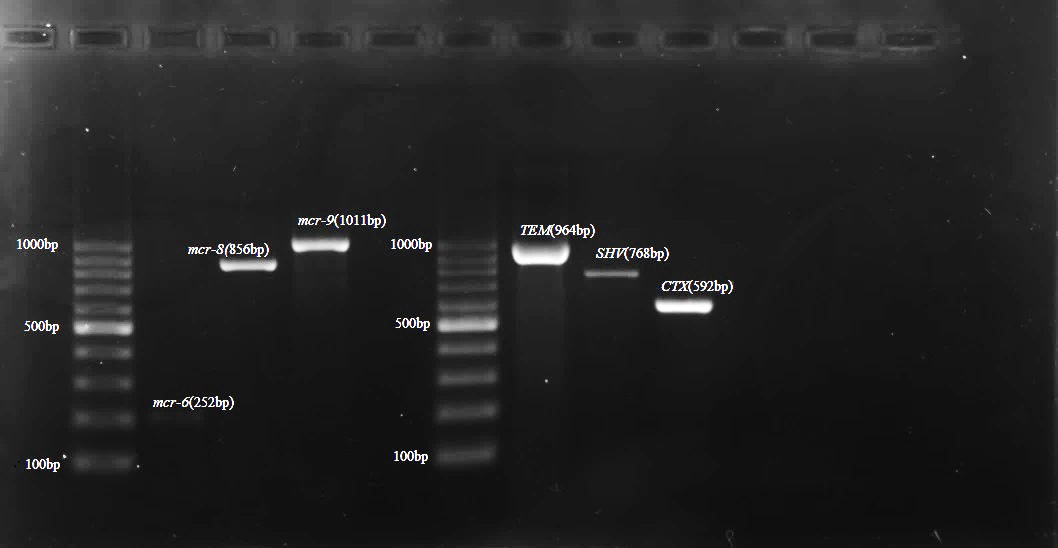


Figure 6. Original multiplex PCR6-9, gel image of *mcr-6,mcr-8, mcr-9* and ESBL genes


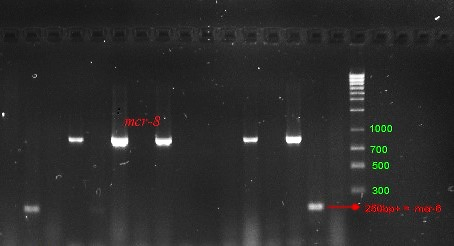


Figure 7. Original gel electrophoresis image of amplified PCR product with mcr-6 and mcr-8 genes


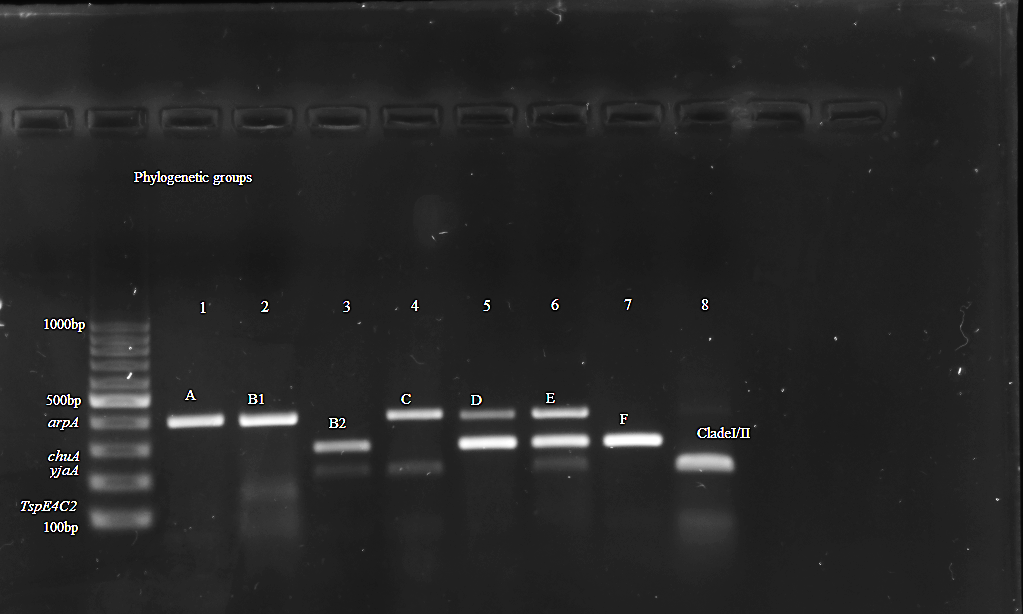


Figure 8. Agarose gel electrophoresis image of mcr positive E. coli phylogenetic typing. Amplified PCR products with E. coli phylogrouping genes; arpA (400bp), chuA (288bp), yjaA (211bp) and TspE4C2 (152bp); lane 1,+ - - -, belonging to phylogroup A; lane 2,+ - - +, belonging to group B1; lane 3,- + + -, group B2; lane 4,+ - + -, group C; lane 5,+ + - -, group D; lane 6,+ + + -, group E; lane 7,- + - - ,group F; lane 8,- - + -, clade I/II.
